# Temporal trends in motor vehicle fatalities in the United States, 1968 to 2010 - a joinpoint regression analysis

**DOI:** 10.1186/s40621-015-0035-6

**Published:** 2015-03-17

**Authors:** Priti Bandi, Diana Silver, Tod Mijanovich, James Macinko

**Affiliations:** Department of Nutrition, Food Studies, and Public Health, Steinhardt School of Culture, Education and Human Development, New York University, 411 Lafayette St, 5th Floor, New York, NY 10003 USA

## Abstract

**Background:**

In the past 40 years, a variety of factors might have impacted motor vehicle (MV) fatality trends in the US, including public health policies, engineering innovations, trauma care improvements, etc. These factors varied in their timing across states/localities, and many were targeted at particular population subgroups. In order to identify and quantify differential rates of change over time and differences in trend patterns between population subgroups, this study employed a novel analytic method to assess temporal trends in MV fatalities between 1968 and 2010, by age group and sex.

**Methods:**

Cause-specific MV fatality data from traffic injuries between 1968 and 2010, based on death certificates filed in the 50 states, and DC were obtained from Centers for Disease Control and Prevention Wide-ranging Online Data for Epidemiologic Research (CDC WONDER). Long-term (1968 to 2010) and short-term (log-linear piecewise segments) trends in fatality rates were compared for males and females overall and in four separate age groups using joinpoint regression.

**Results:**

MV fatalities declined on average by 2.4% per year in males and 2.2% per year in females between 1968 and 2010, with significant declines observed in all age groups and in both sexes. In males overall and those 25 to 64 years, sharp declines between 1968 and mid-to-late 1990s were followed by a stalling until the mid-2000s, but rates in females experienced a long-term steady decline of a lesser magnitude than males during this time. Trends in those aged <1 to 14 years and 15 to 24 years were mostly steady over time, but males had a larger decline than females in the latter age group between 1968 and the mid-2000s. In ages 65+, short-term trends were similar between sexes.

**Conclusions:**

Despite significant long-term declines in MV fatalities, the application of Joinpoint Regression found that progress in young adult and middle-aged adult males stalled in recent decades and rates in males declined relatively more than in females in certain age groups. Future research is needed to establish the causes of these observed trends, including the potential role of contemporaneous MV-related policies and their repeal. Such research is needed in order to better inform the design and evaluation of future population interventions addressing MV fatalities nationally.

**Electronic supplementary material:**

The online version of this article (doi:10.1186/s40621-015-0035-6) contains supplementary material, which is available to authorized users.

## Background

Motor vehicle (MV) fatalities are the leading cause of death in older children, teenagers, and young adults and are one of the top ten leading causes of death for nonelderly adults in the US. (National Highway Traffic Safety Administration 2012). MV crashes, both fatal and nonfatal, impose large economic and social costs from lost productivity, medical costs, and lost quality of life, amounting to about $871 billion in 2010 (Blincoe et al. 2014).

**Figure 1:**
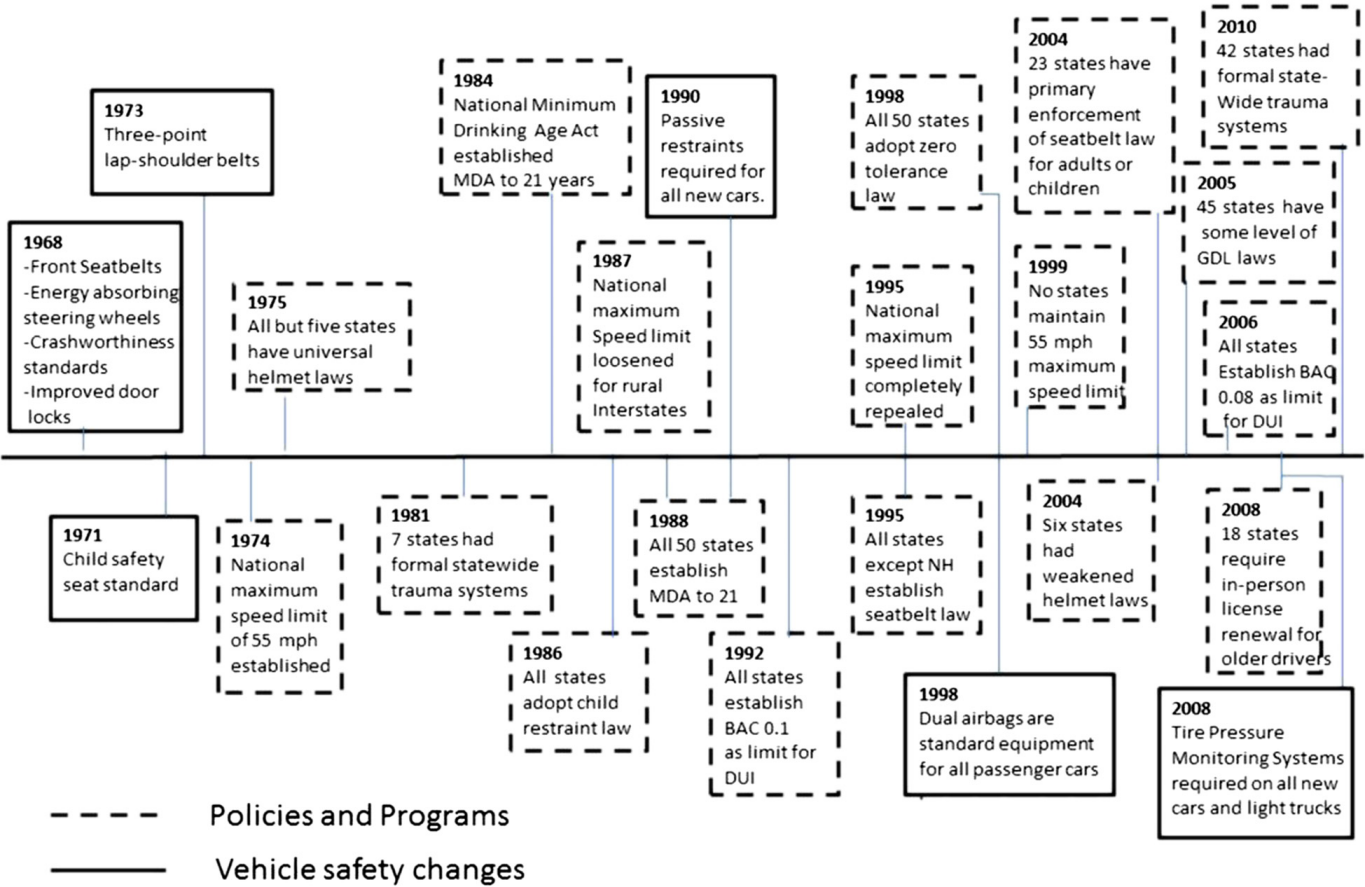
**Selected motor vehicle policy and safety changes in the US, 1968 to 2010.** BAC: Blood Alcohol Concentration; DUI: Driving Under the Influence; GDL: Graduated Driving Licensing; MDA: Minimum Drinking Age; NH: New Hampshire.

Beginning in the last half of the twentieth century, a variety of efforts were introduced to reduce MV fatalities in the US (Figure 1) (Centers for Disease Control and Prevention 1999a). Among these were public health policies and programs targeting individual risk behaviors (alcohol-impaired driving, seatbelt use, speeding, distracted, or drowsy driving) (Task Force on Community Preventive Services 2010; National Highway Traffic Safety Administration 2011) and improvements in vehicle safety design (restraint systems, safety devices, crashworthiness) (National Highway Traffic Safety Administration 2004), roadway infrastructure (Congressional Budget Office 2011; U.S. Department of Transportation 2013), and trauma care (establishment of organized statewide trauma systems). (MacKenzie et al. 2003). Apart from efforts to impact MV fatalities, other secular changes in population demographic and socioeconomic composition, traffic patterns, and exogenous economic and geopolitical events occurred during this time that might have influenced MV fatalities. While there is extensive evidence of the effectiveness of many of these policy and technological efforts in reducing MV fatalities, other changes during this time might have affected progress in reducing MV fatalities, such as repeals of effective laws or the proliferation of car types with high crash fatality risks (Carter et al. 2014; Keall and Newstead 2008; Daly et al. 2006; Trowbridge et al. 2007). Few studies, however, have assessed long-term national temporal trends in MV fatalities in the context of the many complementary and competing factors that occurred during this time. Studies of temporal trends in MV fatalities in the US are limited to evaluations of earlier time periods (Li et al. 2001), and short time spans (Vaca and Anderson 2009) or have focused on specific populations (Vaca and Anderson 2009) or states or regions (Lopez-Charneco et al. 2011; Dischinger et al. 2013; Mitchell et al. 2000). It is important to study long-term national trends not only because it allows for the assessment of progress towards national public health goals but also because it helps identify population subgroups that might not have shared in the progress equally. In fact, given that interventions were often targeted at particular population subgroups (e.g., zero tolerance laws or graduated driving laws for young drivers, child restraint laws for children, or licensure requirements for older adults), it can be hypothesized that differential rates of progress may have occurred in different population age and sex subgroups. Also, given the variety of factors that might have impacted MV fatalities and because many of these factors varied in the timing and uptake across states and localities, it can also be expected that progress over time was not constant. The complexity of this landscape necessitates analytic methods that are able to identify and quantify differential rates of change over time and differences in patterns of trends between population subgroups.

One such analytic method that allows for the identification and measurement of differential rates of change over time is joinpoint regression. Joinpoint regression identifies the points (years) in which there were statistically significant differences in the trend of a chosen outcome and quantifies the short-term increase or decrease between two successive points of change (Kim et al. 2000). Joinpoint regression is particularly appropriate in studying MV fatality trends because it allows for a mechanistic evaluation of changes in long-term trends that may have been affected by numerous complementary or competing factors as detailed earlier. Joinpoint regression has previously been applied to study trends in motor vehicle fatalities in other contexts (Orsi et al. 2012; Richter et al. 2005), but to our knowledge, none have applied this method systematically as a surveillance tool to assess to long-term trends in motor vehicle fatalities in the US. We apply joinpoint regression to assess MV fatality trends in the US in all ages and stratified by age and sex between 1968 and 2010. We discuss our findings in the context of relevant contemporaneous factors that might have impacted MV trends.

## Methods

### Data

Data on MV fatalities came from the Compressed Mortality File (CMF), accessed via the Centers for Disease Control and Prevention Wide-ranging Online Data for Epidemiologic Research (CDC WONDER) online database, a national mortality and population database from years 1968 to 2010 (Centers for Disease Control and Prevention 2014a, 2014b). Mortality data in the CMF is from all death certificates filed in the 50 states and Washington DC and provided to the National Vital Statistics System. Motor vehicle traffic accidents (codes E810-E819) were chosen as the underlying cause of death coded on death certificates according the eighth revision of the International Classification of Disease (ICD-8) prevalent between 1968 and 1978 and the ninth revision (ICD-9) in use between 1979 and 1998. Starting in 1999, the ICD-10 classification system implemented major changes in the coding of injury-related deaths (Anderson et al. 2001). For compatibility with previous classification systems, the following underlying causes of death were coded as motor vehicle deaths from traffic injuries (Miniño et al. 2006) and included pedestrians (V02-V04(.1, .9), V09.2), pedal cyclists (V12-V14(.3-.9), V19(.4-.6)), motorcyclists (V20-V28(.3-.9), V29(.4-.9)), occupants (V30-V79(.4-.9), V83-V86(.0-.3)), other specified (V80(.3-.5), V81.1, V82.1), and unspecified (V87(.0-.8), V89.2). The population estimates are based on official Bureau of the Census estimates of resident populations estimated either via linear interpolation (1968 to 1969), modified census counts from years in which the decennial censuses were conducted, or from intercensal estimates for years between the decennial censuses (Centers for Disease Control and Prevention 2014a). Standard errors for MV fatality rates were also from the CDC WONDER online database and were estimated assuming that the death distribution follows a Poisson distribution (Centers for Disease Control and Prevention 1999b, 2014a). Estimates on vehicle miles traveled (VMT) were obtained from the US Federal Highway Administration and are derived based on two sources of data: annual average daily traffic (AADT) by road segment from the Highway Performance Monitoring System and state-level monthly traffic counts from automatic traffic recorders (Federal Highway Administration 2014).

### Measures

The primary measure of interest was the national MV fatality rate, i.e., MV deaths/100,000 population, for each year between 1968 and 2010. Fatality rates were obtained for males and females separately. The rate for all ages combined was age-adjusted to the 2000 US standard population using 11 age groupings (Centers for Disease Control and Prevention 2014a). In addition, age group-specific fatality rates were obtained for four age groups, including children and young adolescents (<1 to 14 years), older adolescents and early-young adults (15 to 24 years), young and middle-aged adults (25 to 64 years), and older adults (≥65 years). These groups broadly represent age categories that have different levels of risk behaviors that were the subject of targeted policies. Data on annual VMT, a measure of the amount of driving, are used to depict change in exposure to the risk for death from a motor vehicle crash during this time (Beck et al. 2007). Adjustment for VMT is conducted in order to obtain estimates of population risk rather than burden (deaths/population) that are comparable across time or across populations. In sensitivity analyses, we estimated an alternate MV fatality rate, by using annual VMT as the denominator instead of population counts. However, since annual VMT data is not available by age-sex subgroups, we could only estimate this measure for all ages combined.

### Statistical analysis

Temporal trends were analyzed using joinpoint regression, a statistical method that fits a series of joined straight lines between statistically significant changes in trend (joinpoints) and estimates the change between joinpoints using NCI’s Joinpoint Regression Program Version 4.1.1 (Joinpoint Regression Program 2014; Kim et al. 2000; National Cancer Institute 2014).

The generalized log-linear joinpoint regression model for the observations: (*x*_1_, *y*_1_),…, (*x*_*N*_, *y*_*N*_), where *x*_1_ < … < *x*_*N*_ represent the time variable, e.g., calendar year, and *y*_*i*_, *i* = 1, 2, …, *N* represents the annual rates is as follows (Kim et al. 2000): log (*y*_*i*_) = *E*[*y*_*i*_|*x*_*i*_] + *ε*_*i*_, where *ε*_*i*_ is the residual for the *i*th time, and the regression mean *E*[*y*_*i*_|*x*_*i*_] is defined as a succession of (*n* + 1) linear segments over the time interval [*a*,*b*]: *E*[*y*_*i*_|*x*_*i*_] = *β*_0_+ *β*_1_*x*_*i*_ + *δ*_1_ (*x*_*i*_−*τ*_1_)^+^ + … + *δ*_*n*_ (*x*_*i*_−*τ*_*n*_)^+^, where (*x*_*i*_−*τ*_*k*_)^+^ = (*x*_*i*_−*τ*_*k*_) if (*x*_*i*_−*τ*_*k*_) > 0 and 0 otherwise. *δ*_*n*_ is the difference between slopes of the (*k* + 1)th and *k*th segment and the *τ*_*k*_, *k* = 1, 2, …*n*, *n* < *N* is the *k*th unknown joinpoint or statistically significant change in trend with *τ*_0_ = a and *τ*_*n+*1_ = *b*.

In our analyses, *x*_*i*_ represented years between 1968 and 2010 and *y*_*i*_ is the annual MV fatality rate during this time. The final model is a series of joined log-linear segments between successive joinpoints, with each segment described by its short-term annual percentage change (APC). The APC between two successive joinpoints *τ*_*k*_ and *τ*_*k+*1_ was estimated as (*e*^(*β*1+*δ*1+…*δk*)^−1)*X*100 (Kim et al. 2000). The estimated APCs are scale-invariant and can therefore be compared across population subgroups with very different fatality rates. Long-term trends over the entire time series are average annual percentage changes (AAPCs) and were estimated as the weighted average of the short-term APCs, with the weights equal to the length of the short-term line segment (Clegg et al. 2009).

The following parameter settings were specified in the models fit in our analyses. Random errors were assumed to be heteroscedastic (have nonconstant variance), and regression coefficients were estimated by weighted least squares. Analyses corrected for autocorrelation of random errors using weighted least squares based on autocorrelation parameter estimated from the data. However, since correcting for autocorrelation this way within the Joinpoint Program can lead to a loss in power to detect joinpoints, we present sensitivity comparison against uncorrelated errors models (Kim et al. 2000; National Cancer Institute 2014). The grid search method, with 0 grid points (meaning that joinpoints can occur exactly at the observed years), was chosen to search for the location of the joinpoints (years); this method creates a ‘grid’ of all possible locations for joinpoints and tests the sum of squared errors for each one to find the best possible fit.

To determine the optimal number of joinpoints, the model selection method used sequential permutation tests. Each one of the permutation tests performs a test of the null hypothesis *H*_0_: number of joinpoints = *k*_*a*_ against the alternative *H*_*a*_: number of joinpoints = *k*_*b*_ where *k*_*a*_ *< k*_*b*_. The procedure begins with *k*_*a*_ = MIN or minimum number of joinpoints and *k*_*b*_ = MAX or maximum number of joinpoints, in our cases 0 and 5, respectively. Monte Carlo simulation, with the number of permutations set to 4,499, is used to calculate the permutation *p* value for each hypothesis test. The *p* value is compared against the significance level adjusted for overall over-fitting error probabilities, where the adjusted *α* (*k*_*a*_; *k*_*b*_) = *α*/(MAX−*k*_*a*_). If the null is rejected, then *k*_*a*_ is increased by one, otherwise, *k*_*b*_ is decreased by one. The procedure continues until *k*_*a*_ = *k*_*b*_, and the final value of $\widehat{k}$ = *k*_*a*_ = *k*_*b*_ is the selected number of joinpoints. Based on the Joinpoint Regression Program’s recommendations for the number of time points of observations in our study, our analyses allowed a maximum of five joinpoints meaning that between one and six trend segments could be in the final model depending on the number of joinpoints detected.

In the results, first, we present long-term trends in MV fatalities in terms of AAPCs for all ages combined and for each age group, for males and females separately. Age and sex differences in AAPCs were considered statistically significant if the 95% confidence intervals around the estimates did not overlap. Next, we describe short-term trends (APCs) for all ages combined and for each age group, for males and females separately. Two-sided *t*-tests at *p* < 0.05 were used to test whether AAPCs and APCs were significantly different from zero. Those that were significantly different from zero are described with the terms ‘increased’ or ‘decreased’, and nonsignificant trends are described either as ‘stable’, ‘level’, ‘nonsignificant increase,’ or ‘nonsignificant decrease’. We then present a sensitivity comparison to models assuming uncorrelated random errors.

## Results

### Joinpoint analyses of long-term trends - average annual percent change (AAPC)

Long-term trends (AAPCs) between 1968 and 2010 in age-adjusted and age category-specific MV fatality rates, in males and females, are shown in Table 1. Between 1968 and 2010, on average, the MV fatality rate in all ages declined significantly by 2.4% per year in males from 42.7 to 15.4 per 100,000 and by 2.2% per year in females from 14.8 to 6.3 per 100,000. Rates in males and females in all four age groups during the study period declined significantly. The largest average annual declines between 1968 and 2010 were observed in those aged <1 to 14 years in both males (4% per year from 12.1 to 2.2 per 100,000) and females (3.6% per year from 7.5 to 1.8 per 100,000). Among the remaining age groups in males, mortality decreased the most annually between 1968 and 2010 in those aged 15 to 24 years (3% per year from 78 to 22.3 per 100,000), followed by those 65 years and over (2.4% per year from 57.7 per 100,000 to 20.5 per 100,000), and those aged 25 to 64 years (2% per year from 41.8 to 17.6 per 100,000), but differences were not statistically significant. In females, all three age groups 15 years or older had similar rates of decline; between 1968 and 2010, in females aged 15 to 24 years, rates declined by 2% per year from 21.4 to 9.6 per 100,000, in those aged 25 to 64 years by 2.1% per year from 14 to 6.3 per 100,000, and in those aged 65 years and over by 2% from 24.2 to 10.8 per 100,000. Differences in long-term AAPCs between males and females were not significant in any age group.

**Table 1:** **Joinpoint analysis of long-term trends in motor vehicle fatalities rates, all ages, by sex, 1968 to 2010**

	**Males**	**Females**
**Age group**	**AAPC**	**AAPC**
	**% (95% CI)**	**% (95% CI)**
<1 to 14 years	−4.0 (−4.6, −3.3)*	−3.6 (−4.3, −2.9)*
15 to 24 years	−3.0 (−3.7, −2.2)*	−2.0 (−2.6, −1.4)*
25 to 64 years	−2.0 (−2.6, −1.4)*	−2.1 (−2.8, −1.5)*
≥65 years	−2.4 (−3.2, −1.7)*	−2.0 (−2.8, −1.2)*
*All ages (age-standardized)*	*−2.4 (−2.9*, −1.8)*	*−2.2 (−2.8*, −1.6)*

Models are based on random errors corrected for autocorrelation estimated from the data by the Joinpoint Program. JP, Joinpoint; AAPC, Modify to average annual percent change; CI, confidence interval; *Two-tailed significant at *p* < 0.05.

### Joinpoint analyses of short-term - annual percent change (APC)

#### All ages

**Figure 2:**
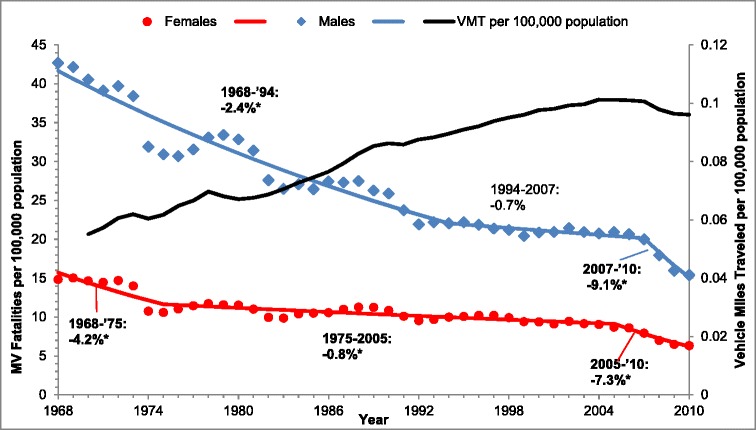
**Joinpoint analysis of trends in motor vehicle traffic fatality rates, all ages, by sex, 1968 to 2010.** Percent depicted in figure are Annual Percent Changes (APCs) from Joinpoint analysis of age-standardized MV fatalities trends. Models are based on random errors corrected for autocorrelation estimated from the data by the Joinpoint Program. *APC is two tailed significant at p<0.05; MV- Motor vehicle.

Figure 2 shows the short-term trends (APCs) in the age-adjusted MV fatality rates in all ages, in males and females between 1968 and 2010. Additional file 1: Table S1 provides these estimates with 95% confidence intervals. Annual number of miles traveled in motor vehicles (vehicle miles traveled or VMT) per 100,000 population is plotted on the secondary axis in Figure 2 to depict the changing levels of motor vehicle travel or driving exposure during this time. Based on multiple permutation tests that kept the overall level of type I error to less than 0.05, the final model selected for males detected 2 joinpoints in 1994 and 2007 and 2 joinpoints for females in 1975 and 2005. In males, the rate declined significantly by 2.4% per year between 1968 and 1994 followed by a stall in decline until 2007 (1994 to 2007: −0.7%). In females, after a significant decline of 4.2% per year between 1968 and 1975, a steady and significant decline of 0.8% per year was observed between 1975 and 2005. Starting in the mid-2000s, fatality rates in both males (2007 to 2010: −9.1% per year) and females (2005 to 2010: −7.3% per year) declined sharply and significantly until 2010.

#### Age categories

**Figure 3:**
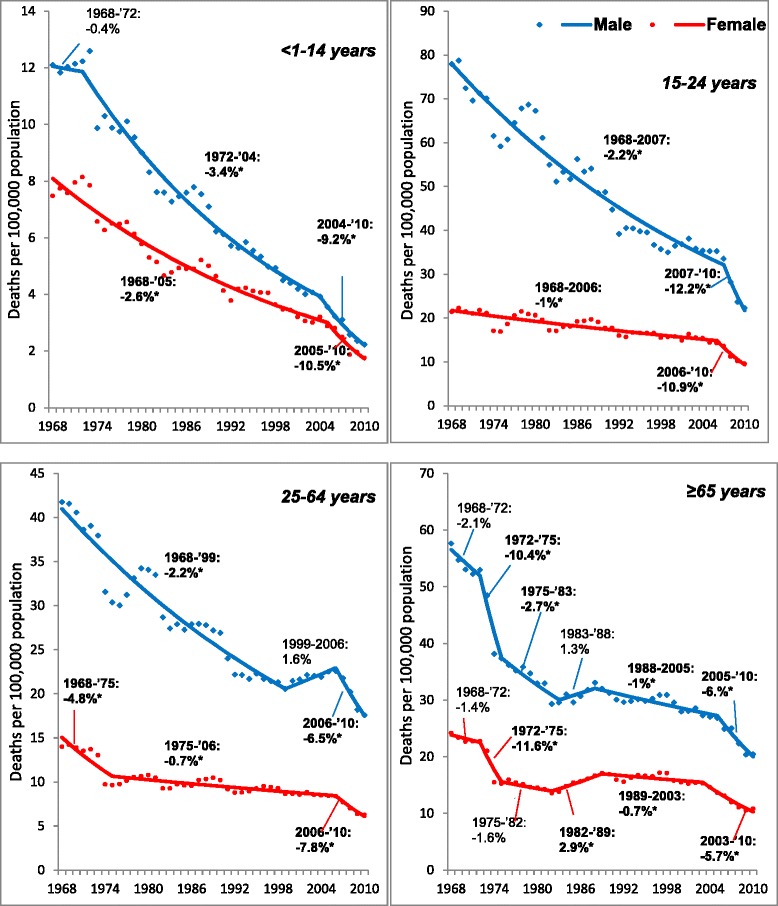
**Joinpoint analysis of motor vehicle traffic fatality trends, by age groups and sex, 1968 to 2010.** The Y-axis scale varies across age group figure panels. Percentages depicted in figure s are Annual Percent Changes (APCs) from Joinpoint analyses of age-standardized MV fatalities trends Models are based on random errors corrected for autocorrelation estimated from the data by the Joinpoint Program. *APC is two tailed significant at p<0.05; MV- Motor vehicle.

Figure 3 (panels 1 to 4) shows the short-term trends (APCs) in the age category-specific MV fatality rates, in males and females between 1968 and 2010. Additional file 1: Table S1 provides these estimates with 95% confidence intervals.

##### <1 to 14 years

Based on multiple permutation tests that kept the overall level of type I error to less than 0.05, the final model selected for males detected 2 joinpoints in 1972 and 2004 and 1 joinpoint for females in 2005. Trends in males and females were generally similar. After a nonsignificant annual increase between 1968 and 1972, the rate in males declined significantly by 3.4% per year between 1972 and 2004. In females, the rate declined significantly by 2.6% per year between 1968 and 2005. Starting in the mid-2000s, sharp, significant declines were observed in both males (2004 to 2010: −9.2% per year) and females (2005 to 2010: −10.5% per year).

##### 15 to 24 years

Based on multiple permutation tests that kept the overall level of type I error to less than 0.05, the final model selected for males detected 1 joinpoints in 2007 and 1 joinpoint for females in 2006. Males and females had similar pattern of trends. MV fatality rates declined significantly between 1968 and the mid-2000s, but declines were significantly larger in males (1968 to 2007: −2.2% per year, 95% CI: −2.5%, −2.0%) than in females (1968 to 2006: −1% per year, 95% CI: −1.2%, −0.8%). Subsequently, rates in both males (2007 to 2010: −12.2% per year) and females (2006 to 2010: −10.9% per year) experienced significant sharp and comparable declines.

##### 25 to 64 years

Based on multiple permutation tests that kept the overall level of type I error to less than 0.05, the final model selected for males detected 2 joinpoints in 1999 and 2006 and 2 joinpoints for females in 1975 and 2006. Males and females had differing patterns of trends over the study period. Between 1968 and 1999, the rate in males declined significantly by 2.2% per year and was subsequently followed by a nonsignificant annual increase of 1.6% until 2006. In contrast, in females, after a significant short-term decline of 4.8% per year between 1968 and 1975, rates experienced a long-term significant decline of 0.7% per year until 2006. Between 2006 and 2010, the rate declined significantly by 6.5% per year in males and by 7.8% per year in females.

##### ≥65 years

Based on multiple permutation tests that kept the overall level of type I error to less than 0.05, the final model selected for males detected 5 joinpoints in 1972, 1975, 1983, 1988, and 2005 and 5 joinpoints for females in 1972, 1975, 1982, 1989, and 2003. In both males and females, a nonsignificant annual decline was observed between the 1968 and 1972 followed by a sharp significant decline until 1975 (males: −10.4% per year, females: −11.6% per year). Subsequently, starting in 1975 and continuing to 1983, the rate continued to decline significantly but at a slower rate of 2.7% per year in males but declined nonsignificantly in females. Between 1982 and 1989, the rate increased significantly by 2.9% per year in females but increased nonsignificantly in males between 1983 and 1988. Between 1988 and 2005, the rate decreased significantly by 1% per year in males and in females by 0.7% per year between 1989 and 2003. Starting in 2005 for males and in 2003 for females, the rate declined significantly by 6% and 5.7% per year until 2010, respectively.

### Sensitivity analyses comparing to uncorrelated errors model

Additional file 2: Table S2 shows the joinpoint analyses assuming uncorrelated errors of long-term (AAPCs) and short-term trends (APCs) in the age-adjusted and age category-specific MV fatality rates, in males and females between 1968 and 2010. Sensitivity checks were conducted because correcting for potential autocorrelation as we have in our main analyses can result in a significant loss of power in detecting joinpoints. As expected, more joinpoints were detected in all ages combined, ages 15 to 24 years and 25 to 54 years when uncorrelated errors were assumed. However, the main findings remained the same in all ages combined and for ages 25 to 64 years, except for a series of nonsignificant increases and decreases during the 1970s based on joinpoints detected in 1972, 1975, and 1979. In males 15 to 24 years, however apart from the joinpoints detected during the 1970s (1975, 1978), the uncorrelated error model detected a stall in the rate of decline in males between 1998 and 2006 similar to that observed in all males and 25- to 64-year-old males. Results for age categories <1 to 14 years and ≥65 years were similar to the autocorrelated error models.

## Discussion

This analysis of long-term trends in MV fatalities in the US found that there have been significant declines in both males and females in all age groups between 1968 and 2010. A unique finding from the application of Joinpoint Regression was that the 42-year time period was characterized by short-term changes in trend for all ages combined, and age groups representing young and middle-aged adults (25 to 64) and older adults (≥65 years). Moreover, the pattern of short-term trends differed between males and females in all ages combined and those ages 25 to 64 years. Whereas in males, large declines between the 1968 and the mid-to-late 1990s were followed by a stall until the mid-2000s, rates in females experienced a long-term steady decline during this entire period that was smaller than those observed in males. Trends in those <1 to 14 years and 15 to 24 years were mostly constant over time, but the magnitude of decline was larger in males than in females in the latter age group.

While there may be several reasons for the observed trends in MV fatalities, there is extensive evidence linking adoption of policies targeting MV-related risk behaviors to reductions in MV fatalities (Task Force on Community Preventive Services 2010; National Highway Traffic Safety Administration 2011). Mainly beginning in the 1980s, several major federal and state policy efforts have continued to varying degrees until present (Figure 1). The sharp declines in males of all ages, and specifically adolescents, young adults, and middle-aged adult males (15 to 24 years, 25 to 64 years), until the mid-to-late 1990s correspond to the period of increased alcohol-related laws targeted at these age groups, including minimum drinking age laws and zero tolerance laws establishing legal blood alcohol concentration (BAC) levels in youth, and with laws establishing progressively lower BAC levels in adults (National Highway Traffic Safety Administration 2014). However, the stall or reversal of declining trends in males starting in the mid-to-late 1990s corresponds to a repeal of some state laws with proven effectiveness in reducing MV fatalities (National Highway Traffic Safety Administration 2006, 2011, 2013; Hedlund 2013). For example, following the 1995 repeal of the national maximum speed limit, states began to raise speed limits such that by 1999, no state maintained the 55-mph speed limit on rural interstates and 34 states have raised speed limits to 70 mph or higher on some part of their roadway systems (Governors Highway Safety Association 2014). Similarly, following the lifting of federal sanctions against states without universal helmet laws in 1995, six states weakened their state helmet laws (Centers for Disease Control and Prevention 2012). Research has indicated that the repeal of these laws was associated with increases in MV fatalities (Houston and Richardson 2007; Richter et al. 2005; Friedman et al. 2009; Strom et al. 2013). The continuation of the stagnation of MV fatality trends into the mid-2000s indicates that the gains that could have been had in young drivers from the passage of zero tolerance and graduated driver laws in the late 1990s and in adults from stronger BAC laws through the mid-2000s were potentially negated by weakening policies with demonstrated population benefits. The observed MV fatality trends in children and older adults are more difficult to place within the context of policy efforts explicitly targeting these groups. In the case of children, despite the demonstrated effectiveness of several child passenger safety technological innovations in reducing child MV fatalities, their adoption into statutory law across states was slow and uneven (Bae et al. 2014). On the other hand, in the case of older adults, policy innovations specifically targeting this age group have been few and largely restricted to state licensure requirements. However, there is lesser evidence regarding the effectiveness of these laws in reducing MV fatalities in this age group, except for in-person renewal requirements (Grabowski et al. 2004; Morrisey and Grabowski 2005).

There are other reasons not related to policy efforts that could have potentially contributed to the observed trends. For example, there have been dramatic improvements in automobile safety, including the introduction of passenger car airbags, safety belts, shatter resistant windshields, etc. (Centers for Disease Control and Prevention 1999a Congressional Budget Office 2011). At the same time, highway infrastructure funding increased tremendously with attendant improvements such as better delineation of road curves, improved illumination, and addition of barriers separating oncoming traffic lanes, etc. (Centers for Disease Control and Prevention 1999a). Another reason could be the changes in the amount of driving during this time. Our analyses did not adjust for vehicle miles traveled, an indicator for exposure to the risk for death from a motor vehicle crash (Beck et al. 2007). It has been noted that the number of miles traveled in motor vehicles or VMT per 100,000 population had been consistently increasing through most of the last half of the twentieth century (Figure 1) (Puentes and Tomer 2008; Polzin 2006). Starting in the early 2000s, however, the growth in VMT per 100,000 population began to stabilize and then decline in the mid-2000s. Since our analyses did not adjust for driving exposure (see ‘Strengths and limitations’ section for sensitivity checks), part of the declines observed in MV fatalities, including the sharp decline observed in all age-sex groups starting in 2006, might correspond to the decline in driving exposure.

This analysis found that the period of increasing policy activity in the 1980s and 1990s corresponded to a larger mortality decline in males than in females, especially in older adolescent, young adult, and middle-aged adult groups. Some of the observed relatively lower decline in females compared to males may be because of the reported larger increase in driving exposure in females than in males (Federal Highway Administration 2011; Insurance Institute for Highway Safety 2012). On the other hand, declines in females may have been lower because laws implemented or repealed during this time addressed risk factors for MV injuries that are more prevalent in males than in females. It is known that female drivers are less likely to engage in risky behaviors, such as speeding and alcohol-impaired driving, that can influence crash risk and case fatality from crashes (Kelley-Baker and Romano 2010; Liu et al. 2005; Elliott et al. 2006). However, this study indicates that policies, while conceived as sex neutral in that they are intended to have similar impacts in males and females, may have different population-level effects because of the gendered nature of the risk behaviors such policies are targeting, On the other hand, studies have shown that females may have responded more rapidly to public health policy and technological innovations targeting MV fatalities, including campaigns to promote seatbelt use and reduce alcohol-impaired driving (Waldron et al. 2005). Given that the population burden of MV-related deaths is much higher in males, arguments can be made for the legitimacy of the observed gendered differences. However, it is important that future research understand the causes of the relatively lower decline in females and their implication for policies and interventions to reduce MV fatalities in the US.

### Strengths and limitations

The joinpoint regression method is a useful and feasible method to understand trends in MV traffic fatality analyses. Our analyses showed that while a straightforward long-term analysis of trend between two time periods (1968 and 2010) would demonstrate that rates in all age-sex groups experienced significant long-term declines in the study period, the joinpoint regression method allowed for the identification of distinct patterns of short-term trends that evidently differed between males and females in certain age groups. The method, already widely used in cancer trend analyses, has significant analytic advantages for injury surveillance and epidemiology.

However, the application of the joinpoint method to MV fatalities was not without limitations. The results were sensitive to parameter specifications including the choice of assuming autocorrelated vs. uncorrelated random errors. Sensitivity analyses comparing our models to models assuming uncorrelated random errors found that our models detected fewer joinpoints in ages 15 to 24 and 25 to 54 years, but overall results remained the same for most age groups. However, in 15- to 24-year-old males, the autocorrelated error model did not detect the stall in decline between the late 1990s to the mid-2000s and so might have been prone to a loss of power in detecting significant changes in trend. Our choice to present autocorrelation-corrected models was conservative, and readers must note that the stall in decline in MV fatalities observed in those 25 to 64 years might have also occurred in males 15 to 24 years. Another limitation is that we did not adjust for VMT, a measure of driving exposure. As a sensitivity check, we conducted a joinpoint regression analysis on MV fatality trends for all ages combined that adjusted for VMT by constructing an outcome of age-adjusted MV fatality rate per VMT. The VMT-adjusted analyses detected 4 joinpoints (1976, 1979, 1992, and 2007) compared to 2 joinpoints in the unadjusted model (1994 and 2007) (results not shown, available from corresponding author). Unfortunately, since VMT is not available by age or sex, we could not adopt the VMT-adjusted MV fatality rate as the main outcome in this study.

The ICD system that provides guidelines for classification of cause of death underwent three changes during the period of study. The change from ICD-9 to ICD-10 in 1999 represented a major change in the classification of injury-related mortality, which could impact the consistency of measurement of MV deaths between ICD periods (Anderson et al. 2001; Miniño et al. 2006). However, the compatibility ratio for the classification of MV deaths between ICD-9 and ICD-10 is 0.9545 indicating that this classification change might have a minimal impact on MV fatality trends (Anderson et al. 2001).

## Conclusions

MV fatalities declined significantly in the US between 1968 and 2010 in all age and sex groups. The application of the joinpoint regression method, however, showed that this decline was marked by short-term changes in trend that included stagnation in progress in recent years in young adult and middle-aged males. It is important to identify reasons for the stall nationally, including the role of contemporaneous repeals of evidence-based policies such as speed limits and universal helmet laws. In addition, much of the declines observed contemporaneously with policy efforts in adolescents, young, and middle-aged adults occurred to a larger degree in males than in females. Future research must consider the observation of sex differences in progress in the design and evaluation of population-based efforts to reduce MV fatalities.

## **Additional files**

Additional file 1: Table S1. Joinpoint analysis of motor vehicle traffic fatality trends, all ages, and by age groups and sex, 1968 to 2010. Additional file 2: Table S2. Joinpoint analysis of motor vehicle traffic fatality trends assuming uncorrelated random errors, all ages, and by age groups and sex, 1968 to 2010.

## Abbreviations

AAPC: average annual percentage change
APC: annual percentage change
BAC: blood alcohol concentration
CDC: Centers for Disease Control and Prevention
CMF: Compressed Mortality File
ICD: International Classification of Diseases
MV: motor vehicle
VMT: vehicle miles traveled
